# Notification of malaria cases in the Brazilian Amazon Basin from 2010 to 2020: an analysis of the reporting times

**DOI:** 10.1186/s12936-023-04464-y

**Published:** 2023-02-10

**Authors:** Mario J. C. Ayala, Naiara C. M. Valiati, Leonardo S. Bastos, Daniel A. M. Villela

**Affiliations:** 1 Programa de Computação Científica, Fundação Oswaldo Cruz (Fiocruz), Rio de Janeiro, Brazil; 2 Politécnico Grancolombiano, Escuela de Optimización, Diseño y Automatización, Bogotá, Colombia

**Keywords:** Malaria, Reporting times, Health surveillance

## Abstract

**Background:**

As controlling malaria transmission remains a public-health challenge in the Brazilian Amazon basin, the National Surveillance System for Malaria (SIVEP-MALARIA) has registered malaria notifications for over fifteen years helping in the decision-making on control and elimination. As a surveillance database, the system is prone to reporting delays, and knowledge about reporting patterns is essential in decisions.

**Methods:**

This study contains an analysis of temporal and state trends of reporting times in a total of 1,580,617 individual malaria reports from January 2010 to December 2020, applying procedures for statistical distribution fitting. A nowcasting technique was applied to show an estimation of number of cases using a statistical model of reporting delays.

**Results:**

Reporting delays increased over time for the states of Amazonas, Rondônia, Roraima, and Pará. Amapá has maintained a similar reporting delay pattern, while Acre decreased reporting delay between 2010 and 2020. Predictions were more accurate in states with lower reporting delays. The temporal evolution of reporting delays only showed a decrease in malaria reports in Acre from 2010 to 2020.

**Conclusion:**

Malaria notifications may take days or weeks to enter the national surveillance database. The reporting times are likely to impact incidence estimation over periods when data is incomplete, whilst the impact of delays becomes smaller for retrospective analysis. Short-term assessments for the estimation of malaria incidence from the malaria control programme must deal with reporting delays.

## Background

Malaria transmission remains a health challenge in the Americas despite the decrease in malaria burden during the 21st century. Brazil had the second highest malaria incidence in this region in 2020 [1]. Brazil implemented the Epidemiological Surveillance System for Malaria (SIVEP-Malaria) that registers all malaria cases, and this system constitutes a critical tool in the Brazilian control and elimination programme [2]. Notifying Units (NU) report the malaria cases to the Municipal Health Departments (SMS) that provide malaria reports to the SIVEP-Malaria, and this process can take several days in the Amazon Basin region, which accounts for 99% of malaria cases in Brazil [3, 4]. The delay in notifications between the eventual malaria cases and the reported cases in the surveillance system implies a challenge in the elimination programme, lagging responses to unusual transmission trends [5].

A comprehensive analysis of notification periods over time and across the states that are part of the Amazon basin is lacking in understanding how malaria surveillance provides timely information. Previous works have implemented technological and mathematical tools for predicting malaria cases using previous surveillance and climatic and socioeconomic data. Quan et al. implemented a technical framework of surveillance through nurse smartphones in South Africa following malaria reports at the time [6]. A set of models implemented statistical tools such as regressive models with time-series, nonlinear models, and spatio-temporal models for estimating malaria cases using meteorological data and previous malaria reports for predicting high-risk areas and alerting for possible outbreaks in some countries of Africa and Asia [7–10]. Further, a set of previous works have analysed and predicted notification delays of malaria in Guyana and South Africa, correcting reporting delays using a nowcasting framework and an autoregressive time-series [11, 12].

Here, the reporting times of malaria notifications were analysed with secondary data from the Brazilian surveillance database (SIVEP-malaria). The distribution of reporting times was fitted to statistical distributions for exploring reporting delays of malaria in the Brazilian Amazon basin across states and over time. A Bayesian hierarchical model was applied to demonstrate the effect of correcting (nowcasting) the reporting delays as Bastos et al. [13].

## Methods

### Epidemiological data

A dataset of 1,580,617 individual malaria reports from January 2010 to December 2020 in the Brazilian Amazon region was obtained from the Brazilian Information System of Epidemiological Surveillance (SIVEP). This dataset accounts for the state, municipality, notification date, symptoms date, and reporting date [4]. The reporting delays were calculated by the difference between the reporting date and notification date using the individual reports from the states of Amazonas (AM), Acre (AC), Roraima (RR), Rondônia (RO), Pará (PA), and Amapá (AP). As a result, 82,172 reports were excluded by surpassing 182 days of delay (unusual reports), and 70 were excluded by inconsistent dates, i.e., later notification date.

### Distribution fitting

Different distributions were fitted to data of reporting times by each state per year, estimating distribution parameters, such as means and standard deviations. This approach allows exploring the changes in reporting delay by state per year. The tested distributions were Weibull and Gamma for fitting all reporting delays due to the proficiency of these distributions for describing distributions with positive skewness and kurtosis as the empirical distributions of reporting delay.

The distributions were fitted by maximum likelihood estimation function (MLE) $L(\theta )$ using a parametric distribution $f(.|\theta )$ with parameters $\theta \in {{\mathbb {R}}}$ (Eq. 1):

1 $$\begin{aligned} L(\theta )= \prod _{i=1}^{n} f(x_i|\theta ), \end{aligned}$$

for *n* observations $x_i$ of reporting times and $f(.|\theta )$ distribution function.

The numerical analysis involved Nelder-Mead, quasi-Newton and conjugate-gradient algorithms for the distribution fitting of the MLE function. The implementation involved the fitdistr function with optim function in R to obtain $\theta$ parameters of $f(x_i|\theta )$ that maximize MLE function [16, 17]. Therefore, we fitted 66 sets of parameters with these distributions describing reporting delays for six states (AM, AC, RR, RO, AP, and PA) per 11 years (2010-2020). The MLE function obtained shape and scale parameters for Weibull distribution, and shape and rate parameters for Gamma distribution by each state.

### Bayesian model for predictions

The Bayesian model from Bastos et al. [13] was applied for correcting reporting delays, estimating the random variable $n_{t,d}$ that accounts for the number of malaria events occurred at time $t=1,2,...,T$, but reported with delays of $d=1,2,...,D$ time units [13], where *T* represents the time step with data availability and *D* represents the maximum allowed delay. $n_{t,d}$ was modelled using a negative binomial distribution with mean $\lambda _{t,d}$ and scale $\phi$ (see Eq. 2)

2 $$\begin{aligned} n_{t,d} \sim NegBin(\lambda _{t,d},\phi ) \qquad \lambda _{t,d}>0,\phi >0. \end{aligned}$$

The choice of prior distributions for $\phi$ was exponential distribution *Exp*(0.1) with mean 10 and standard deviation 10. The expression for $log(\lambda _{t,d})$ captures temporal variability (see Eq. 3):

3 $$\begin{aligned} log(\lambda _{t,d})=\mu +\alpha _t+\beta _d+\gamma _{t,d}+\eta _{w(t)}, \end{aligned}$$

where $\mu$ is the overall mean at the log-scale, $\alpha _t$ is the random effect that captures the mean structure of temporal evolution, $\beta _d$ is the random effect that captures the mean structure of delay, and $\gamma _{t,d}$ is the random effect that captures the time-delay interaction. $\alpha _t$, $\beta _d$ and $\gamma _{t,d}$ were modelled using random walks in order 1 (see Eq. 4, 5 and 6).

4 $$\begin{aligned} \alpha _t \sim N(\alpha _{t-1},\sigma _\alpha ^{2}), \qquad t=2,3,...,T. \end{aligned}$$

5 $$\begin{aligned} \beta _t \sim N(\beta _{t-1},\sigma _\beta ^{2}), \qquad d=2,3,...,D. \end{aligned}$$

6 $$\begin{aligned} \gamma _{t,d} \sim N(\gamma _{t-1,d},\sigma _\gamma ^{2}). \end{aligned}$$

Half normal HN($\tau ^2$) prior distributions were assumed for $\sigma _\alpha$, $\sigma _\beta$ and $\sigma _\gamma$ using the means in logarithm scale $\tau =1$ for $\beta _d$, and $\tau =0.1$ for $\alpha _t$ and $\gamma _{t,d}$. $\eta _{w(t)}$ is the random effect that captures the weekly seasonal component with $w(t)=1,...,52$ weeks. Parameter $\eta _{w(t)}$ is defined as a second-order random effect (see Eq. 7) adopting a half normal HN(1) prior distribution with $\tau =1$ for $\eta _{w(t)}$.

7 $$\begin{aligned} \eta _{w} \sim N(2\eta _{w-1}-\eta _{w-2},\sigma _\eta ^{2}). \end{aligned}$$

The estimation procedure with the Bayesian model is implemented adopting the Integrated Nested Laplace Approximation (INLA) with INLA package in R [18, 19]. The value of *T* is generally applied to the epidemiological week of interest for prediction and $D=26$, except stated otherwise.

## Results

**Fig. 1:**
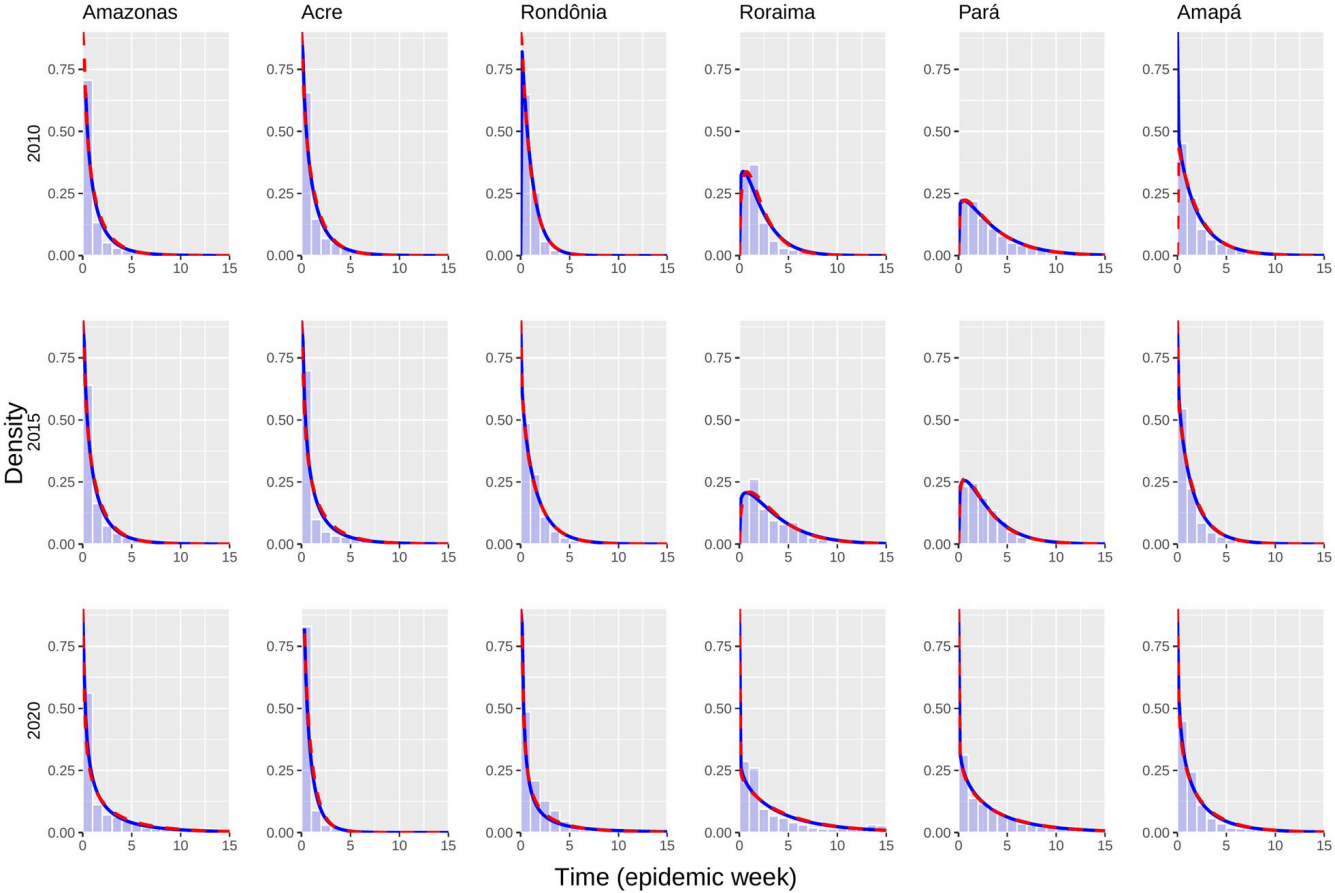
Delay-distribution fitting per State during 2010, 2015, and 2020. Complete blue lines represent Weibull distribution fitting, dotted red lines represent Gamma distribution fitting, and bars represent real data

Reporting times varied between 0 and 16 weeks with 99% confidence in all states in the Amazon basin. Delay distributions in Acre (AC) and Amazonas (AM) presented most of the reports in less than two weeks (see Fig. 1). Delay distributions in Roraima (RR), Rondônia (RO), Pará (PA), and Amapá (AP) presented more variability than AC and AM, with delays over two weeks with higher frequencies. Also, delay distributions in RR, PA, and RO also showed more variability in 2020 than in previous years. Delay distribution in AM and AP presented a similar variability over the years, and RR presented distributions moved to the right, evidencing an increase in reporting delay. The distribution in AC in 2020 moved to the left, implying a reduction in reporting delays in this state.

**Fig. 2:**
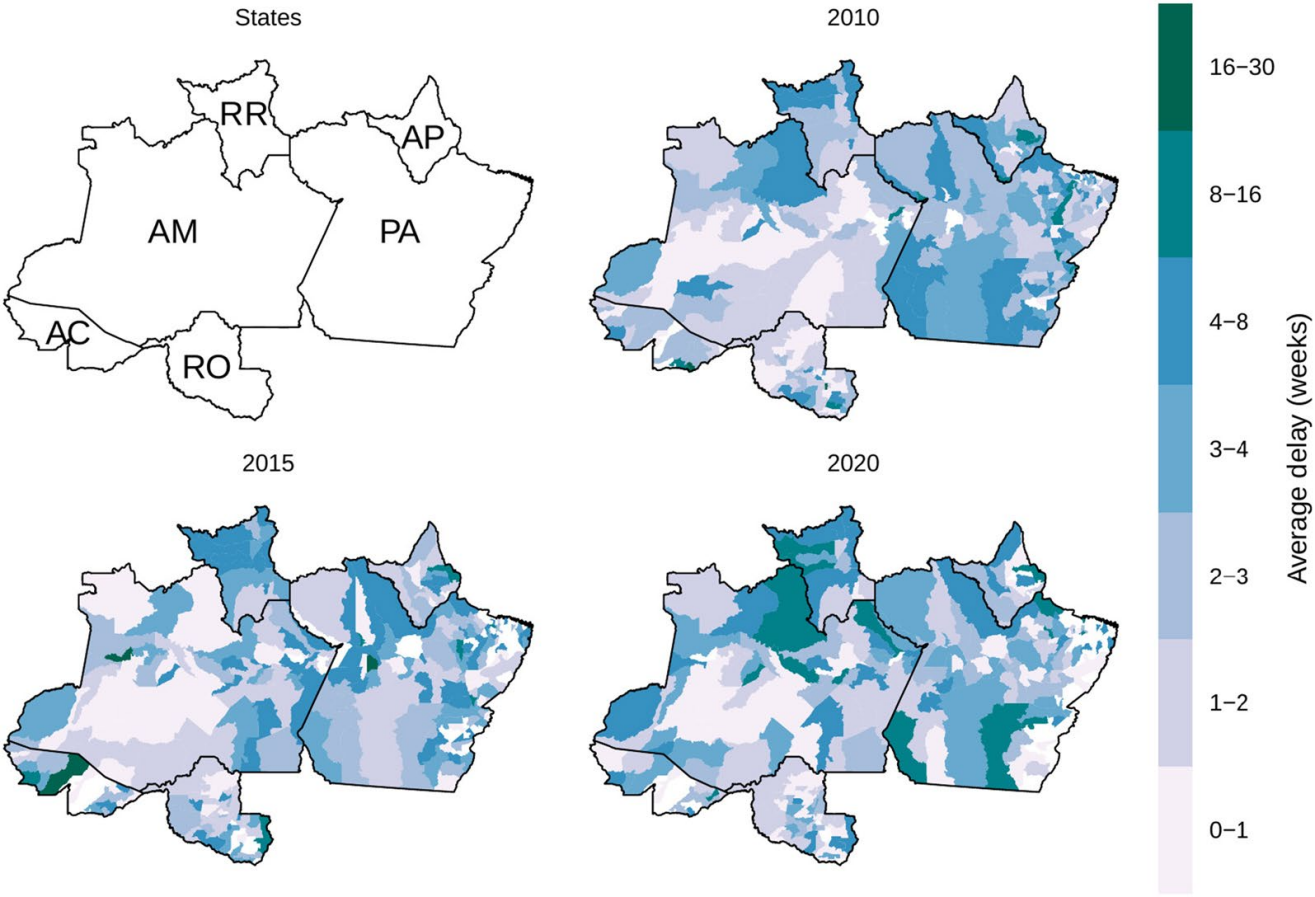
State units of the Brazilian Legal Amazon and average delays in weeks per municipality in years 2010, 2015 and 2020

The average reporting delays were above eight weeks in several municipalities between 2010, 2015, and 2020 (Fig. 2). Average delays above eight weeks were mainly in RR, northwest of AM, south, and northwest of PA, and west of AP in 2020. RR evidenced an increase in the average delay in multiple municipalities from 2010 to 2015. AM also evidenced an increase in the average delay in the municipalities in the northwest region. Although average delays decreased in PA from 2010 to 2015, some municipalities increased the average delay in 2020. PA also evidenced an increase in municipalities without malaria reports (white regions) in the west of PA in 2020 despite the increment in the average delay in the other municipalities of this state. AC did not exhibit a decrease in the average delay in all points. Still, the average delays decreased in the east region (Mâncio Lima and Cruzeiro do Sul), linking with the results in Fig. 1, because the east region accounts for most of the malaria cases in AC. RO and AP evidenced high delays in the western regions, and AP evidenced an increase compared from 2010 to 2015.

**Fig. 3:**
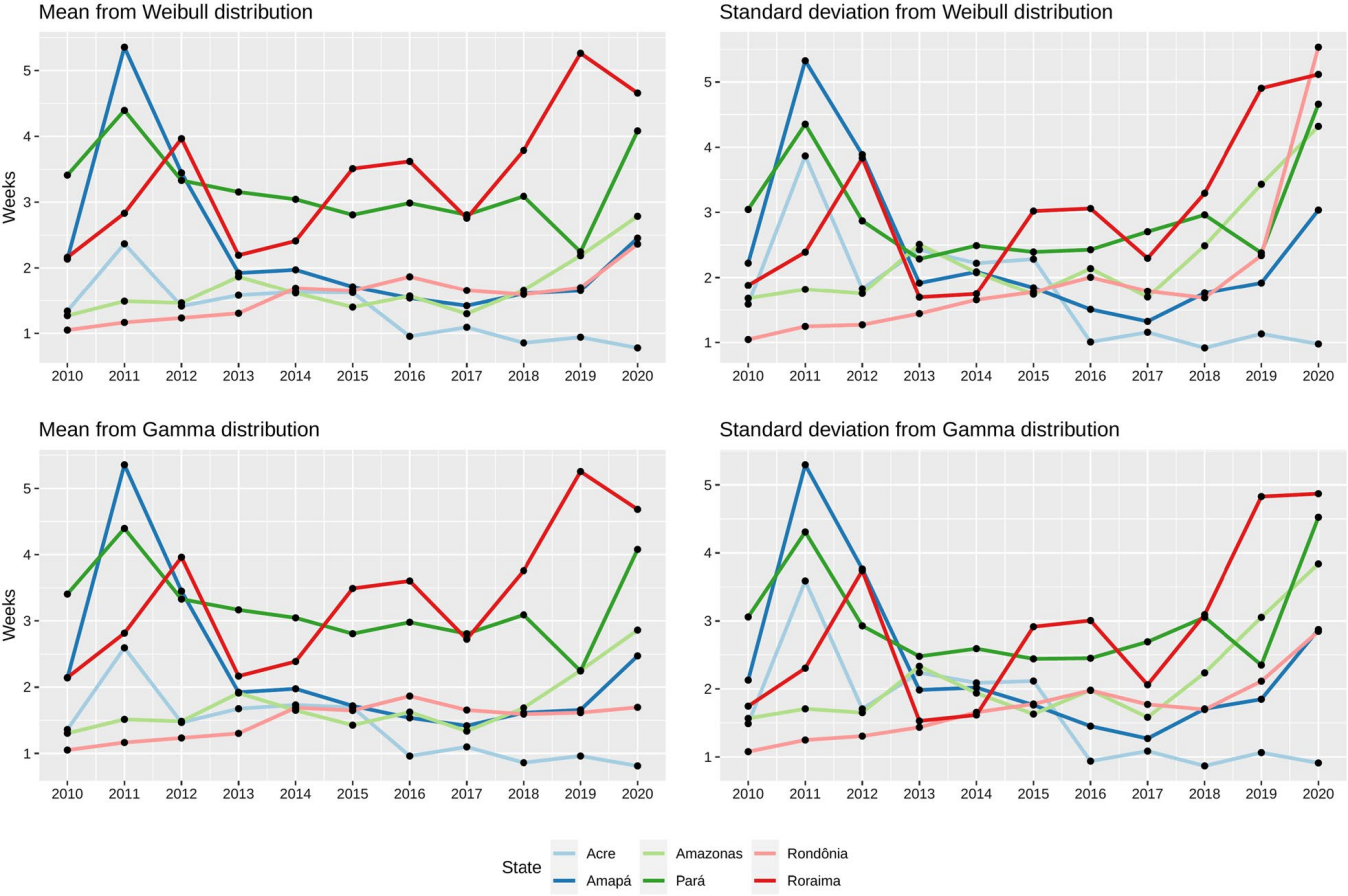
Mean and standard deviation of reporting delay from fitted distributions per year

A similar pattern of reporting-delay distribution is observed in AM, AC, RO, and AP states with positive asymmetry and skewness coefficients illustrating the tendency to low delays using Weibull and Gamma distributions (see Figs. 1 and 3). This pattern of distribution fitting was also found in RR and PA, only in 2020, because the skewness of the distribution observed in these states was different in 2010 and 2015. Distributions only exhibited a decrease in the delay mean and standard deviation in AC, evidencing a decline in reporting delays in that state. Delay distributions in AM and RO indicated increases in the mean and standard deviation of delays across the time at these states. Delay distributions in RR and PA also exhibited increases in delays’ mean and standard deviation. Still, these states presented a greater increase in the mean and standard deviation compared to other ones. Finally, the delay distribution in AP exhibited a similar mean and standard deviation in 2010 and 2020 and a decrease in the mean and standard deviation in 2015.

**Fig. 4:**
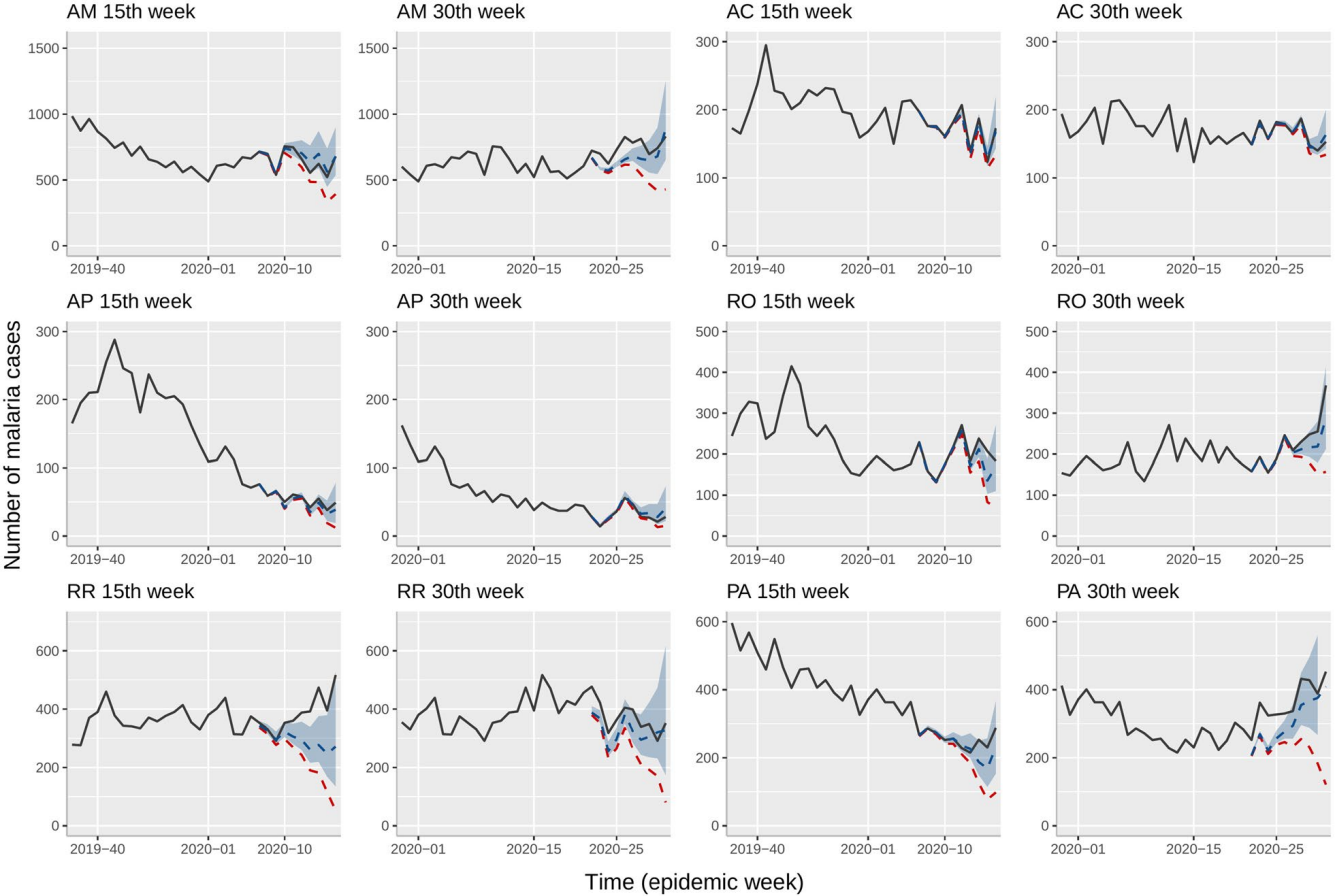
Model predictions in 2020. This figure illustrates model predictions in the 15th and the 30th epidemiological weeks in 2020. These weeks represent different seasonal moments. Black lines represent the eventually reported cases, red dotted lines represent the current reported cases and blue dotted lines represent model predictions. Light blue regions represent the prediction intervals

The Bayesian model obtained accurate predictions anticipating the malaria reports, mainly in states with lower reporting delays (see Fig. 4). The model obtained the best-adjusted prediction in AC and AP, states those presented the least reporting delays before 15st and 30th epidemiological weeks in 2020. Conversely, the model obtained the least adjusted prediction in RR and PA, which presented the greatest reporting delays. Still, the trends from model predictions cases were similar, as seen in the 30th week for PA. Moreover, the prediction interval permitted estimating a scenario with a higher number of cases that contained the eventual cases illustrating the use of the Bayesian model predicting malaria cases on different levels of reporting delay.

## Discussion

The accumulated reporting delays increased over time for the states of Amazonas, Rondônia, Roraima, and Pará. Amapá has maintained a similar pattern of reporting delay while reporting times decreased in Acre between 2010 and 2020. Nevertheless, the average delays per municipality evidenced an increase in the municipalities with delays above eight weeks in Roraima, Amazonas, and Pará in this period. In general, states with lower reporting delays had more accurate predictions.

The increase in reporting times in most Amazon-basin states, except Acre, evidenced that the surveillance system of Brazil still needs to accomplish international standards. Braz et al. found that recording notification in the Amazon region was below international standards in the timelines in malaria notification between 2003 and 2012, and Fig. 3 illustrates that reporting delay experimented increases the mean and standard deviation in 2020 in comparison to 2010 in most of the states [3]. Although the COVID-19 pandemic in 2020 might also help to enlarge reporting times in the Amazon basin due to the priorities in COVID-19 attention, Fig. 3 illustrates the increases in mean and standard deviation before COVID pandemic [22]. The malaria rebound between 2017 and 2019 might also explain the rise in reporting delay compared to previous years that obtained fewer cases [23]. Geographical exploration of the average reporting times suggests an expected higher reporting times in municipalities located in isolated areas, i.e., far from medium and large cities such as state capitals. The Brazilian Amazon Basin covers a large area with several municipalities with difficult access to notifying malaria cases. More studies with variables describing health access and surveillance limitations can help to determine the impact due to such factors.

Roraima and Pará obtained the highest mean in reporting delay in 2020, implying the least accurate predictions in the Bayesian model, and socioeconomic factors in border areas might explain this situation. The North region of Roraima borders Venezuela and Guyana, and the increase of migrants seeking a job in this region has implied an increase in epidemic peaks, showing the necessity of improving surveillance systems in border areas [21]. One of the principal challenges in malaria elimination and control is human mobility in border areas, and surveillance systems must extend efforts to maintain available data in real-time between countries in the Amazon region [24, 25, 27, 31]. The unexpected increase of malaria cases in Pará from 2016 to 2018 might explain the rise in reporting delays in this region despite the mean and standard deviation of delay in 2019 being the least since 2010 [28].

The malaria surveillance system in Brazil has presented two challenges: recording errors and reporting delays. A previous study in the state of Amazonas showed that epidemic fluctuations influenced recording errors, and our predictive model might help to estimate a more accurate report during malaria epidemics [20]. On the other hand, the priorities of notification agents in other activities, the bureaucratic fill-in process, some missing attributes in the reports, and erroneous data in patient declaration might increase the delay in the notification process, impacting data quality [3]. Likewise, an anticipated reporting of malaria cases from the current model might help to establish rapid priority control and elimination plans such as vector control [26]. In fact, predictions from the model output are generally close to the final number of cases, even though the model achieved more accurate predictions with low reporting delays, as the time series in Acre showed. The random effects indirectly capture possible heterogeneities due to organizational difficulties in reporting, even without a particular variable to describe such factors.

The prediction from the model implementation is based on previous and current reports using interactions with the seasonal behavior of malaria, time-independent behaviour, reporting delay behaviour, and time-delay interaction. The analysis structure is based on the Bastos et al. model that joins the interactions with co-variables and the effect of the spatial distribution of delays for dengue and Severe Acure Respiratory Infections in public surveillance databases [13]. Likewise, Tigis et al. found spatial heterogeneity in reporting delays of malaria cases in Guyana, implying the necessity of involving a spatial interaction for obtaining more accurate predictions [29]. On the other hand, Thwing et al. corrected malaria incidence by considering suboptimal rates of care-seeking and testing delays instead of analysing delay interaction over time [30]. Although the current model has not had a spatial interaction and suboptimal reporting rates, this approach can help surveillance efforts since the model only requires previous reports.

Malaria reports in the Brazilian Amazon Basin have presented reporting delays in most states, and the temporal evolution of reporting delays only showed a decrease in malaria reports in Acre from 2010 to 2020. Consequently, assessments from the malaria control programme over short terms must deal with reporting delays (nowcasting). Therefore, modelling tools such as Bayesian models might provide reliable predictions allowing rapid responses to malaria epidemics and improving elimination efforts.

## Data Availability

This study used data from SIVEP-malaria surveillance database per request to the Ministry of Health (N. 25820.002062/2019-41). Requests should be directed directly to the Ministry of Health – https://esic.cgu.gov.br.

## References

[1] WHO. World malaria report 2021 . World Health Organization; 2021. https://apps.who.int/iris/rest/bitstreams/1398397/retrieve. Accessed 1 Mar 2022.

[2] da Silva NS, da Silva-Nunes M, Malafronte RS, Menezes MJ, D’Arcadia RR, Komatsu NT (2010). Epidemiology and control of frontier malaria in Brazil: lessons from community-based studies in rural Amazonia. Trans R Soc Trop Med Hyg.

[3] Braz RM, Tauil PL, Santelli AC, Fontes CJ (2016). Evaluation of the completeness and timeliness of malaria reporting in the Brazilian Amazon, 2003–2012. Epidemiol Serv Saude.

[4] Baroni L, Pedroso M, Barcellos C, Salles R, Salles S, Paixão B (2020). An integrated dataset of malaria notifications in the legal Amazon. BMC Res Notes.

[5] WHO (2012). Disease surveillance for malaria elimination: an operational manual.

[6] Quan V, Hulth A, Kok G, Blumberg L (2014). Timelier notification and action with mobile phonestowards malaria elimination in South Africa. Malar J.

[7] Kim Y, Ratnam JV, Doi T, Morioka Y, Behera S, Tsuzuki A (2020). Publisher correction: malaria predictions based on seasonal climate forecasts in South Africa: a time series distributed lag nonlinear model. Sci Rep.

[8] Bationo CS, Gaudart J, Dieng S, Cissoko M, Taconet P, Ouedraogo B (2021). Spatio-temporal analysis and prediction of malaria cases using remote sensing meteorological data in Diébougou health district, Burkina Faso, 2016–2017. Sci Rep.

[9] Wangdi K, Singhasivanon P, Silawan T, Lawpoolsri S, White NJ, Kaewkungwal J (2010). Development of temporal modelling for forecasting and prediction of malaria infections using timeseries and ARIMAX analyses: a case study in endemic districts of Bhutan. Malar J.

[10] Jones AE, Wort UU, Morse AP, Hastings IM, Gagnon AS (2007). Climate prediction of El Niño malaria epidemics in north-west Tanzania. Malar J.

[11] Menkir TF, Cox H, Poirier C, Saul M, JonesWeekes S, Clementson C (2021). A nowcasting framework for correcting for reporting delays in malaria surveillance. PLoS Comput Biol.

[12] Ebhuoma O, Gebreslasie M, Magubane L (2018). A seasonal autoregressive integrated moving average (SARIMA) forecasting model to predict monthly malaria cases in KwaZulu-Natal South Africa. S Afr Med J.

[13] Bastos LS, Economou T, Gomes MFC, Villela DAM, Coelho FC, Cruz OG (2019). A modelling approach for correcting reporting delays in disease surveillance data. Stat Med.

[14] C. Codeço, F. Coelho, O. Cruz, S. Oliveira, T. Castro, L. Bastos. Infodengue: A nowcasting system for the surveillance of arboviruses in Brazil. Revue d’épidémiologie et de Santé Publique. 2018;66:(5)s386.

[15] Wickham H (2016). ggplot2: elegant graphics for data analysis.

[16] Delignette-Muller ML, Dutang C. "Fitdistrplus: An R Package for Fitting Distributions”. J Stat Software. 2015; 64:(4)1-34.

[17] R Core Team. R: A Language and Environment for Statistical Computing. Vienna, Austria. 2020.

[18] Blangiardo M, Cameletti M, Baio G, Rue H (2013). Spatial and spatio-temporal models with R-INLA. Spa Spatiotemporal Epidemiol.

[19] Rue H, Martino S, Chopin N (2009). Approximate Bayesian inference for latent gaussian models by using integrated nested Laplace approximations. J Royal Stat Soc Ser B.

[20] Wieffels Alexandre, Wolfarth-Couto Bruna, Filizolai Naziano, Durieux Laurent, Manegas Morgan (2016). Accuracy of the malaria epidemiological surveillance system data in the state of Amazonas. Acta Amaz.

[21] Arisco NJ, Peterka C, Castro MC (2021). Cross-border malaria in Northern Brazil. Malar J.

[22] Daboin BEG, Bezerra IMP, Morais TC, Portugal I, Echeimberg JO, Cesar AEM (2022). Deciphering multifactorial correlations of COVID19 incidence and mortality in the Brazilian Amazon Basin. Int J Environ Res Public Health.

[23] Lana R, Nekkab N, Siqueira AM, Peterka C, Marchesini P, Lacerda M (2021). The top 1%: quantifying the unequal distribution of malaria in Brazil. Malar J.

[24] Ferreira MU, Castro MC (2016). Challenges for malaria elimination in Brazil. Malar J.

[25] Recht J, Siqueira AM, Monteiro WM, Herrera SM, Herrera S, Lacerda MVG (2017). Malaria in Brazil, Colombia, Peru and Venezuela: current challenges in malaria control and elimination. Malar J.

[26] Baia-da-Silva DC, Brito-Sousa JD, Rodovalho SR, Peterka C, Moresco G, Lapouble OMM (2019). Current vector control challenges in the fight against malaria in Brazil. Rev Soc Bras Med Trop.

[27] Carlos BC, Rona LDP, Christophides GK, Souza-Neto JA (2019). A comprehensive analysis of malaria transmission in Brazil. Pathog Glob Health.

[28] Ayala MJC, Bastos LS, Villela DAM (2022). On multifactorial drivers for malaria rebound in Brazil: a spatio-temporal analysis. Malar J.

[29] Menkir Tigist F, Cox Horace, Poirier Canelle, Saul Melanie, Jones-Weekes Sharon (2021). A nowcasting framework for correcting for reporting delays in malaria surveillance. PLoS Comput Biol.

[30] Thwing J, Camara A, Candrinho B, Zulliger R, Colborn J, Painter J, Plucinski MM (2020). A robust estimator of malaria incidence from routine health facility data. Am J Trop Med Hyg.

[31] Saldanha R, Mosnier E, Barcellos C, Carbunar A, Charron C, Desconnets J, et al. Contributing to elimination of cross-border malaria through a standardized solution for case surveillance, data sharing, and data interpretation: development of a cross-border monitoring system. JMIR Public Health Surveill. 2020;6(3):15409. 10.2196/15409 PMC7492983 32663141

